# Prize Questions

**Published:** 1860-01

**Authors:** 


PRIZE QUESTIONS.—A prize of one hundred dollars is offered by the Société Medico-Pratique de Paris, for the best paper on the etiology, history, and treatment of Eczema. The statements of the author must be based upon numerous cases. The essays may be written in French or Latin, and must be sent to M. Martin, Hotel de Ville, Paris, before the 31st of December, 1861.

The Medical Society of Amsterdam offers a gold medal, of the value of thirty ducats, for the best essay on the physiology and pathology of Scoliosis. Special attention should be paid to the muscles capable of producing curvature, and to those which may be brought in antagonism with them, and effect a cure. The papers, which should be sent to Dr. J. W. K. Tilanus, of Amsterdam, before the 1st of May, 1861, may be written in any language.

